# Epidemiology and Risk Factors of Wrist Pain and Injury in Adolescent Artistic Gymnasts: A Systematic Review and Meta-analysis

**DOI:** 10.1177/23259671251395329

**Published:** 2026-01-14

**Authors:** Stefania D.F. DiLeo, Atefeh Noori, Erin Day, Timothy A. Burkhart, Ryan A. Paul, Andrea H.W. Chan

**Affiliations:** * Faculty of Kinesiology and Physical Education, University of Toronto, Toronto, Ontario, Canada; † Division of Plastic, Reconstructive and Aesthetic Surgery, Department of Surgery, University of Toronto, Toronto, Ontario, Canada; ‡ Toronto Western Hospital, University Health Network, Toronto, Ontario, Canada; § Temerty Faculty of Medicine, University of Toronto, Toronto, Ontario, Canada; ‖ Division of Orthopaedic Surgery, Department of Surgery, University of Toronto, Toronto, Ontario, Canada; ¶ The Hospital for Sick Children, Division of Orthopaedic Surgery, Toronto, Ontario, Canada; Investigation performed at University of Toronto, Division of Orthopaedic Surgery and Department of Kinesiology and Physical Education, Toronto, Ontario, Canada

**Keywords:** gymnastics, wrist, biology of bone, biomechanics of bone, epidemiology, pediatric sports medicine

## Abstract

**Background:**

Adolescent gymnasts are at increased risk for wrist pathology due to repetitive high-impact wrist loading during the midgrowth spurt.

**Purpose:**

To conduct a comprehensive systematic review on the epidemiology and risk factors for wrist pain and injury in adolescent artistic gymnasts.

**Study Design:**

Systematic review; Level of evidence, 4.

**Methods:**

Under PRISMA (Preferred Reporting Items for Systematic Reviews and Meta-Analyses) guidelines, we searched MEDLINE, EMBASE, CINAHL, Cochrane, and SPORTDiscus from inception to January 2025. We included observational studies of adolescent artistic gymnasts that reported prevalence, incidence, and/or risk factors for wrist pain, acute injuries, chronic injuries (causing physeal stress or not attributable to a specific acute event), or positive ulnar variance (PUV). Where applicable, meta-analyses were conducted to estimate the pooled prevalence and proportion of new cases for each outcome using common- or random-effect models. Results from studies that were not possible to pool were also reported.

**Results:**

Of the 3650 records identified, 25 studies (185,107 gymnasts) were included for analysis. All evidence was deemed low to very low certainty. Nine studies suggested a pooled wrist pain prevalence of 53% (95% CI, 39%-66%), with 1 study reporting the proportion of new wrist pain cases to be 2% over 1 year. The prevalence of acute wrist injuries reported in 1 study was 34%, and the pooled proportion of new cases was 4% (n = 2 studies; 95% CI, 2%-7%) over a 2-year period. The pooled prevalence of chronic wrist injury was 36% (n = 6 studies; 95% CI, 12%-71%), and the pooled proportion of new chronic wrist injury cases was 5% (n = 2 studies; 95% CI, 3%-10%) over a 2-year period. Two studies suggested a pooled PUV prevalence of 4% (95% CI, 1%-14%), with 1 study reporting the proportion of new PUV cases to be 16% over an 18-month period in preteen gymnasts (mean ± SD age, 10 ± 2 years). Wrist pain was significantly associated with age between 10 and 14 years, increased age at training onset, training intensity, body mass index, years training, and weekly training hours.

**Conclusion:**

Our review demonstrated that adolescent artistic gymnasts demonstrate an alarmingly high prevalence of acute and chronic wrist pain and wrist injury. Further research on training intensity thresholds and risk reduction strategies is imperative for informing and implementing guidelines that protect the health of this vulnerable population.

Gymnasts have a high incidence of injuries, with previous reports showing rates up to 9.22 per 1000 athlete-exposures.^28^ Frequently employed as a weightbearing joint, the hyperextended wrist may experience loads up to twice the gymnast's body weight and at loading rates that exceed 16 times one's weight per second.^34^ However, compared with typical weightbearing joints such as the knee, the wrist comprises bones that articulate over smaller contact areas and that contain thinner articular cartilage.^36,38,40,45^ Consequently, wrist pain is a significant concern in gymnasts, with point and lifetime prevalence rates of 88% and 92%, respectively.^33,50^ Wrist injuries are also prevalent, making the wrist the most frequently injured area in male gymnasts and the sixth most injured in their female counterparts.^54^

Key to the success of artistic gymnasts, specifically, is the production of high forces at the hand and wrist while performing on apparatuses such as the floor and vault.^21^ However, for the artistic gymnast in the peak of one's adolescent growth spurt, repeated exposure to such loads may present several consequences to the open physis of the wrist. During this period of rapid skeletal growth, the distal radius and ulnar physes are particularly susceptible to shear and tensile forces that can result in physeal injury, disturbance in chondrocyte mineralization, and longitudinal growth disruption.^15^ This overuse physeal stress injury is otherwise known as the “gymnast wrist” (GW).^41^ GW has previously been cited among the most common causes of wrist pain or injury in gymnastics.^14,23^ GW presents in 3 stages, with stage 1 characterized by wrist pain without radiographic abnormalities.^26^ Stage 2 demonstrates radiographic findings of physeal change indicating stress (chronic) injury, and stage 3 is determined by acquired positive ulnar variance (PUV) or other growth deformity.^26^

Although other high-risk sports have evidence-based guidelines for promoting player safety, including the pitch count in baseball,^32^ concussion guidelines in football,^52^ and neuromuscular training protocols in soccer,^27^ gymnastics lacks similar injury risk reduction guidelines, including that for GW. Increasing awareness of the risk factors associated with wrist pain, injury, and growth disturbances is crucial for developing effective injury risk reduction strategies that promote the health and career longevity of young artistic gymnasts. Previous studies have narratively reported on these outcomes,^8,30^ but a meta-analysis has yet to be conducted. We wanted to provide a comprehensive systematic review and meta-analysis of literature in this population to provide insights into specific wrist injury mechanisms and identify risk factors. The purpose of this study was to present the prevalence and incidence of wrist pain and other findings associated with wrist injury (including GW) in the adolescent artistic gymnast and to identify and evaluate the risk factors that may contribute to its development. We hypothesized that the prevalence and incidence of wrist pain and wrist injury would be high in adolescent artistic gymnasts and that the risk for these outcomes would be increased among gymnasts in the peak of their adolescent growth spurt.

## Methods

Standards for Meta-Analysis of Observational Studies and PRISMA (Preferred Reporting Items for Systematic Reviews and Meta-Analyses) guidelines were followed.

### Literature Search and Study Selection

A literature search was conducted in collaboration with a librarian using MEDLINE, EMBASE, CINAHL, Cochrane, and SPORTDiscus (Appendix Table A1, A [Table table5-23259671251395329][Table table6-23259671251395329][Table table7-23259671251395329]-E). All databases were searched from inception to January 2025 and no limits were applied. Reference lists of eligible studies and related reviews were reviewed for additional articles. The finalized reference list was imported to an online screening platform (Covidence 2024). Two authors (S.D.F.D, E.D.) independently screened the title and abstract to assess studies’ eligibility for inclusion. These 2 authors then independently screened the eligible studies according to their full text. Conflicts present at either stage were resolved by a third author (A.N.).

### Eligibility Criteria

We included observational studies that enrolled an adolescent (≤19 years) artistic gymnast population and reported ≥1 of the following outcomes: prevalence, incidence, and/or risk factors for wrist pain, acute and/or chronic injuries, or PUV in gymnasts. To distinctly evaluate the prevalence and incidence of wrist pathology, studies that reported pain or injury attributed to both the wrist and the hand together or combined acute and chronic types of injuries were excluded. We excluded studies examining adult gymnasts only (>19 years), randomized controlled trials, small case series (N < 20), laboratory-based studies, reviews, posters, or abstracts. We also excluded studies that observed other gymnastic disciplines only (ie, rhythmic, aerobic, acrobatic, or trampoline).

### Data Extraction and Risk-of-Bias Assessment

Two reviewers (S.D.F.D. and E.D.) independently extracted study characteristics, gymnasts’ information (competitive level, start age, weekly training hours, total years training, whether pain/injury affected training, apparatuses causing pain/injury), and reported outcomes among eligible studies. If prevalence rates were not reported directly, the number of gymnasts reported to have pain, injury, or PUV was divided by the total number of gymnasts. The proportion of new cases of wrist injury was collected per total number of gymnasts. Studies that reported the outcomes per total number of injuries were reported separately. Wrist pain was defined as the presence of any type of pain in the wrist not attributed to a specific traumatic event or diagnosed injury at the time of data collection. This definition captured overuse-related or idiopathic pain and was considered distinct from wrist injury, which was defined as a discrete diagnosis. Injuries were categorized as acute or chronic based on the definition provided in the respective study. Acute injuries were those described as either sudden onset injuries resulting in time lost from sport or medical treatment or reports of acute wrist sprain, strain, and/or fracture. Chronic injuries were those described as either overuse physeal stress injuries (ie, GW) or diagnosed injuries not attributable to a specific acute event (with or without time lost from sport). For the second objective, we extracted data related to significant risk factors identified via a regression model.

We evaluated the risk of bias in the eligible studies using the following criteria^11^: (1) the representativeness of the study population—considered high risk if participant recruitment was inadequately described or convenience sampling was used; (2) the validity of the outcome assessment—considered high risk if unvalidated/unreliable instruments (eg, self-report questionnaires) were used; and (3) the amount of missing data—considered high risk if >20% of data were missing at follow-up. For studies that reported risk factors, we also assessed the risk of bias using the following criteria^11^: the control of confounding variables—considered high risk if the study did not adjust for ≥3 potential covariates and the method of statistical analysis—considered high risk if the study did not use a standard method of analysis to reduce bias. The same 2 independent reviewers assessed the risk of bias and discussed with a senior author (A.N.) if discrepancies were raised. The overall risk of bias for each study was summarized as high if any one of the criteria was determined to be high risk.

### Statistical Analysis

We conducted a meta-analysis for each outcome that was reported from 2 studies using the common-effects model (RStudio 2024, Boston, Massachusetts). Outcomes reported from more than two studies were meta-analyzed using the random-effects model. We quantified the pooled prevalence or pooled proportion of new cases along with the associated 95% CI. We conducted the meta-analysis with prevalence estimates that were transformed using the double arcsine method^6^ to avoid the overestimation of weights for studies with estimates toward 100%. We also reported the results of studies that were not possible to pool.

#### Subgroup Analysis

We defined the following a priori subgroup hypotheses to explain heterogeneity, assuming higher rates of wrist pain/injury with (1) female versus male gymnasts; (2) smaller (<60) versus larger (≥60) studies; (3) competitive- versus noncompetitive-level gymnasts (differentiated based on whether gymnasts participated in competitions); and (4) high versus low risk of bias on a criterion-by-criterion basis. Subgroup analyses were conducted if there were ≥2 studies within each category. For all subgroup differences, significance was set at an alpha of .05.

#### Certainty of Evidence

We used the Grading of Recommendations Assessment, Development and Evaluation (GRADE) approach framework to assess the certainty of evidence.^44^ GRADE classifies evidence as high, moderate, low, or very low based on considerations of risk of bias, indirectness, imprecision, inconsistency, and publication bias. We examined heterogeneity for all pooled estimates through visual inspection of forest plots and the *I*^2^. For subgroup analyses based on the risk-of-bias components, if no significant association was found, we included all studies and did not rate down for risk of bias. We assessed the small-study effects using Egger test when ≥10 studies contributed to a meta-analysis. One reviewer (S.D.F.D.) assessed all evaluations, and the final assessment was confirmed by the senior author (A.N.).

## Results

**Figure A1.**
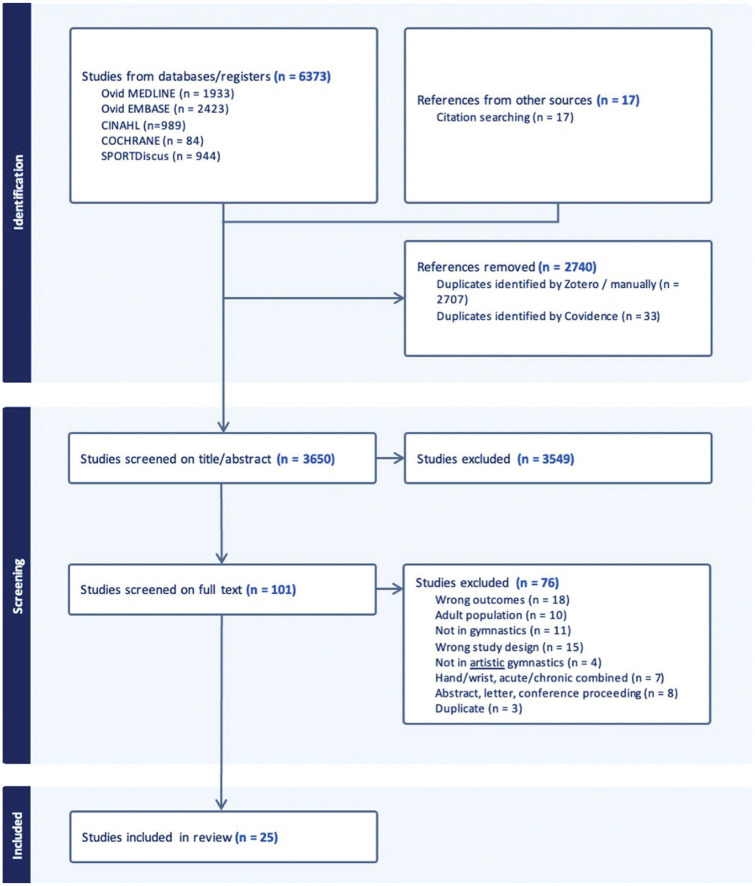
Flow diagram of study selection.

We identified 3650 records after removing duplicates, of which 25 articles met the eligibility criteria (Appendix Figure A1). Two studies^5,19^ were published by the same group of authors and both involved participants from the Junior European Gymnastics Championships. As the study population overlapped, we included the most recent publication^5^ only. The majority of included studies implemented a cross-sectional design (n = 14),^††^ followed by a retrospective design (n = 5),^1,20,37,49,55^ prospective design (n = 5),^2,7,16,31,35^ and case series (n = 1).^41^

Most studies (n = 20)^‡‡^ included competitive-level gymnasts. Two studies^10,46^ included members of a Chinese opera school who performed gymnastics-like training and three^1,49,55^ did not report gymnasts’ competitive level (Table 1). Seven studies^3,15,16,18,22,41,50^ reported the apparatuses on which gymnasts experienced pain or injury. Among the included studies, the sample size ranged from 19 to 183,139 participants and the mean female proportion was 76%. The median age of gymnasts was 12 years (IQR, 11-14). Among the 8 studies in which it was recorded,^2-4,15-17,20,43^ the median body mass index (BMI) was 17 kg/m^2^ (IQR, 17-18). Across studies, the median age at which gymnasts commenced training was 6 years (IQR, 5-7), the median hours of training per week was 17 hours (IQR, 12-22), and the median total years of training was 6 years (IQR, 5-8). Across 6 studies,^3,15,16,18,22,50^ wrist pain and/or injury affected training (ie, limited performance or caused time lost) for 30% to 63% of gymnasts. The method of assessing wrist pain or injury and the time that either was observed varied among the included studies (Appendix Table A2).

**Table 1:** Characteristics of Included Studies^*a*^

General Information						Gymnastics-Related Information					
Author (y)	Study Design (country)	N	Age, y	Female, %	BMI	Competition Level	Start Age, y	Training Hours, h/Week	Total Training Time, y	WP/Injury Affected Training, %	Apparatus Causing WP/Injury
Albright et al (2023)^1^	Retrospective (USA)	183,139	12	86							
Amaral et al (2015)^3^	Cross-sectional (Portugal)	23	11 ± 3	0	17 ± 2	Competitive	6 ± 2	18 ± 4	5 ± 3	30	Pommel horse
Amaral et al (2014)^2^	Prospective (Portugal)	25	10 ± 2	64	17 ± 2	Competitive	5 ± 2	17 ± 3	5 ± 2		
Amaral et al (2012)^4^	Cross-sectional (Portugal)	33	11 ± 2	100	17 ± 2	Competitive	5 ± 1	17 ± 4	6 ± 2		
Aubergé et al (1984)^5 **b**^	Cross-sectional (France)	98		58		Competitive					
Caine et al. (1992)^7^	Prospective (USA)	60	13	65		Competitive					
Chang et al (1995)^10^	Cross-sectional (Taiwan)	261	14 ± 2	55		Gymnastics-like training					
DeSmet et al (1994)^12^	Cross-sectional (Belgium)	191	16	100		Competitive	7	27			
DiFiori et al (2002)^16^	Prospective (USA)	47	10 ± 2	45	17	Competitive	5 ± 2	8 ± 4	4 ± 2	42	Beam, floor, pommel horse
DiFiori et al (2002)^15^	Cross-sectional (USA)	59	9 ± 2	47	17	Competitive	5 ± 2	7 ± 4	4 ± 2	36	Beam, floor, pommel horse
DiFiori et al (1997)^17^	Cross-sectional (USA)	44	12 ± 3	61	18	Competitive	6 ± 2	12 ± 6	5 ± 2		
DiFiori et al (1996)^18^	Cross-sectional (USA)	52	12	62		Competitive	7 ± 2	12 ± 5	5 ± 3	63	Vault, floor, pommel horse, parallel bars
Fari et al (2021)^20^	Retrospective (Italy)	79	14 ± 3	99	18 ± 2	Competitive			7 ± 3		
Guerra et al (2016)^22^	Cross-sectional (Brazil)	19	13	0		Competitive	6	26	8	47	Floor, pommel horse, parallel bars
Konrad and Pförringer (1997)^29^	Cross-sectional (Germany)	289				Competitive		11	9		
Lindner and Caine (1990)^31^	Prospective (Canada)	178		100		Competitive					
McLaren et al (2015)^35^	Prospective (USA)	50	13	100		Competitive			8		
O’Kane et al (2011)^37^	Retrospective (USA)	96	11 ± 2	100		Competitive					
Roy et al (1985)^41^	Case series (USA)	21	12	90		Competitive		27			Beam, vault, floor, uneven bars
Sastre-Munar et al (2022)^43^	Cross-sectional (Spain)	59	17 ± 4	85	21 ± 2	Competitive	7	21 ± 15	10 ± 5		
Shih et al (1995)^46^	Cross-sectional (Taiwan)	47		21		Gymnastics-like training					
Swenson et al (2012)^48^	Cross-sectional (USA)			100		Competitive					
Tisano et al (2022)^49^	Retrospective (USA)										
Trevithick et al. (2018)^50^	Cross-sectional (Australia)	237	15 ± 3	80		Competitive				35	All apparatuses
Wood et al (2010)^55^	Retrospective (Scotland)										
Median (IQR)			12 (11-14)		17 (17-18)		6 (5-7)	17 (12-22)	6 (5-8)		

a Data are presented as mean ± SD where available unless otherwise indicated. BMI, body mass index; WP, wrist pain.

b Includes overlapping data with Duvallet et al.^19^ Only the results of the most recent publication (Aubergé et al^5^) were included.

### Risk of Bias

Among the eligible studies, 80% (20 out of 25)^§§^ were considered to have a high risk of bias in ≥1 criterion. Sixteen studies^‖‖^ failed to recruit a representative population, 11 studies^¶¶^ did not use a valid tool for assessing outcomes, and 5 studies^20,29,31,37,50^ reported a high rate of missing data (Appendix Table A3). Among studies that reported risk factors, 50% (2 out of 4)^20,22^ were considered to have a high risk of bias, with both demonstrating high risk in each of the criteria used (Appendix Table A4).

### Wrist Pain

**Figure A2.**
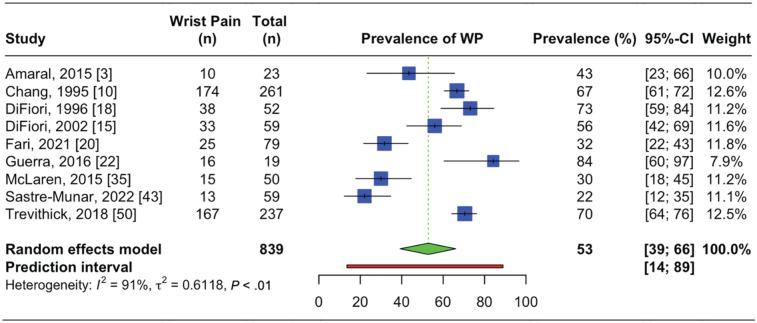
Meta-analysis of the prevalence of wrist pain (WP) in adolescent gymnasts.

**Figure A3.**
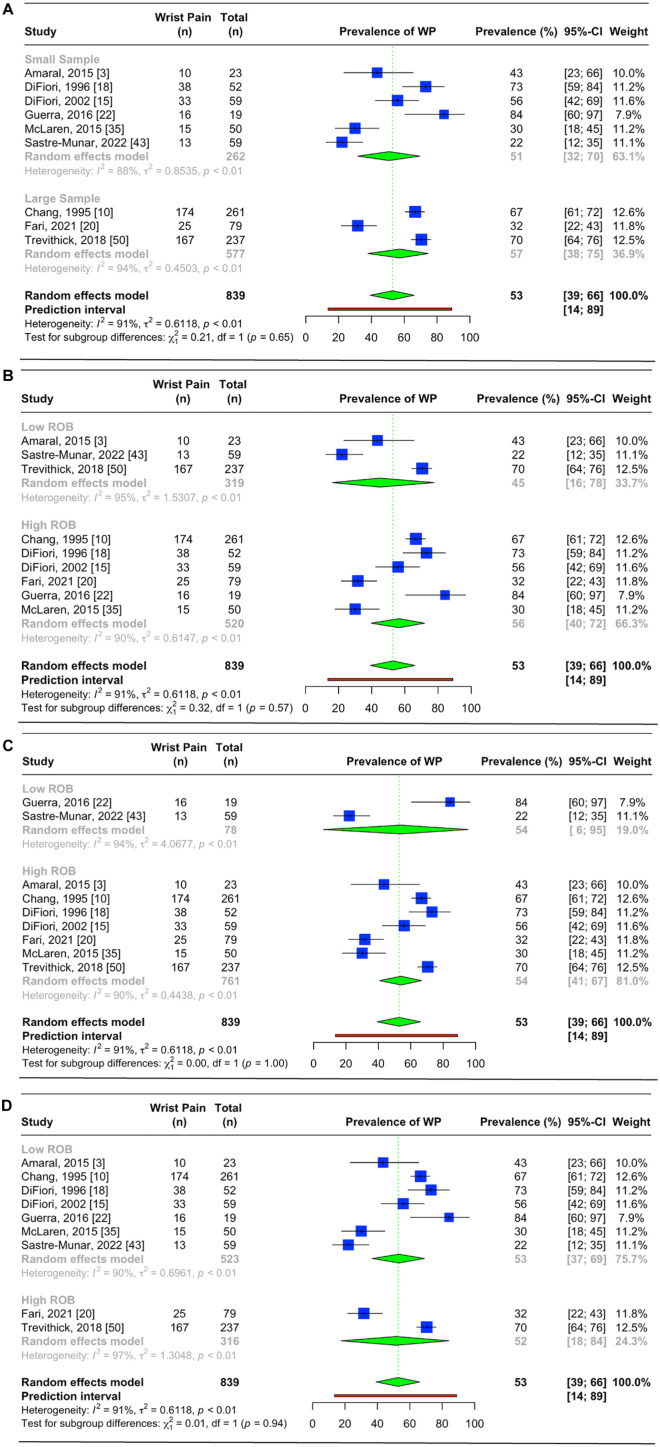
Subgroup analyses for wrist pain (WP) prevalence. (A) Based on small (<60) vs large (≥60) sample size. (B) Based on low vs high risk of bias (ROB) in source population representativeness. (C) Based on low vs high ROB in method of outcome assessment. (D) Based on low vs high ROB in amount of missing data. (A-D) ROB assessed using CLARITY Group criteria.^11^

Very low certainty of evidence from 9 studies^##^ (n = 839 adolescent gymnasts) suggested a pooled prevalence of wrist pain of 53% (95% CI, 39%-66%; *I*^2^ = 91%) (Table 2, Appendix Figure A2). No subgroup analyses were significant (Appendix Figure A3). Only 1 study^16^ reported the proportion of new cases of wrist pain, which was 2% (1/47 gymnasts) over the 1-year study period.

**Table 2:** GRADE Evidence Profile With Summary of Findings^*a*^

Outcome	Studies, n	Gymnasts, n	ROB	Inconsistency	Indirectness	Imprecision	Publication Bias	Effect Estimate, % (95%CI)	Overall Certainty of Evidence
Prevalence of wrist pain	9	839	Not serious^*b*^	Serious^*c*^	Not serious	Serious	Not detected	53 (39-66)	Very low
Prevalence of chronic wrist injury	6	433	Serious	Serious^*c*^	Not serious	Serious	Not detected	36 (12-71)	Very low
Proportion of new cases of acute wrist injury	2	274	Serious	Not serious	Not serious	Not serious	Not detected	4 (2-7)	Low
Proportion of new cases of chronic wrist injury	2	274	Serious	Serious^*c*^	Not serious	Serious	Not detected	5 (3-10)	Very low
Prevalence of PUV	2	56	Serious	Not serious	Not serious	Serious	Not detected	4 (1-14)	Very low

a GRADE, Grading of Recommendations Assessment, Development and Evaluation; PUV, positive ulnar variance; ROB, risk of bias.

b Certainty of evidence was not rated down on the basis of ROB because the subgroup analyses did not show a significant difference between each ROB component and the estimation (Appendix Figure A3, B-D).

c Serious unexplained inconsistency (*I*^2^ = 75%-100%).

### Acute Wrist Injury

Only 1 study^46^ reported the prevalence of acute wrist injury per total number of gymnasts, which was 34% (16/47 gymnasts). Data from 3 studies^29,48,55^ reported that acute wrist injury accounted for between 22% and 46% of the total number of injuries.

**Figure A4.**
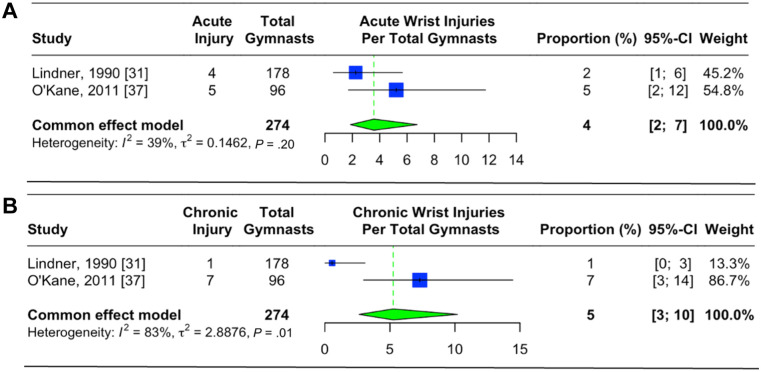
Meta-analysis of the proportion of new cases of wrist injury in adolescent gymnasts. (A) Acute wrist injuries. (B) Chronic wrist injuries.

Low certainty of evidence from 2 studies^31,37^ (n = 274 adolescent gymnasts) suggested a pooled proportion of new cases of acute wrist injuries over a median 2-year period of 4% (95% CI, 2%-7%; *I*^2^ = 39%) (Table 2, Appendix Figure A4A). Additionally, 2 studies not included in the meta-analysis because of insufficient data reported the proportion of new cases of acute injury as 5% over an 8-year period^1^ and 10% over a 7-year period.^49^

### Chronic Wrist Injury

**Figure A5.**
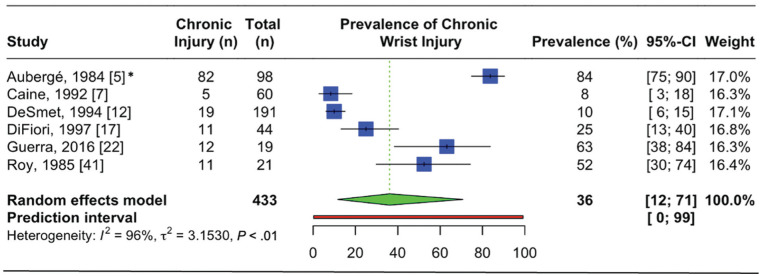
Meta-analysis of the prevalence of chronic wrist injury in adolescent gymnasts. *Contains overlapping data with Duvallet et al.^19^ Only the most recent publication was included in the meta-analysis.

**Figure A6.**
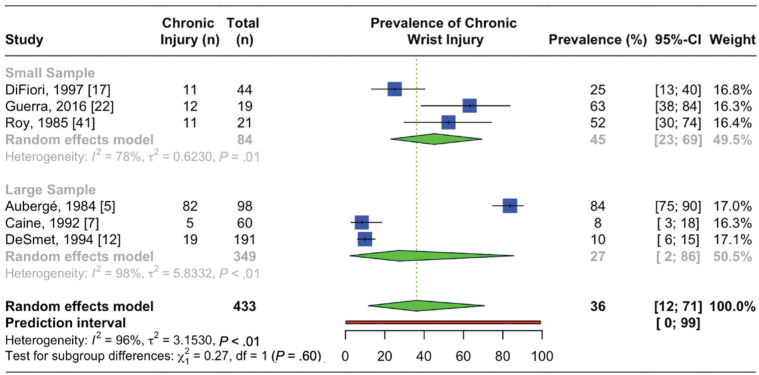
Subgroup analysis for chronic wrist injury prevalence based on small (<60) vs large (≥60) sample size.

Very low certainty of evidence from 6 studies^5,7,12,17,22,41^ (n = 433 adolescent gymnasts) suggested the pooled prevalence of chronic wrist injury was 36% (95% CI, 12%-71%; *I*^2^ = 96%) (Table 2, Appendix Figure A5). No subgroup analyses were significant (Appendix Figure A6).

Very low certainty of evidence from 2 studies^31,37^ (n = 274 adolescent gymnasts) suggested a pooled proportion of new cases of chronic wrist injuries over a median 2-year period of 5% (95% CI, 3%-10%; *I*^2^ = 83%) (Table 2, Appendix Figure A4B).

### Positive Ulnar Variance

**Figure A7.**
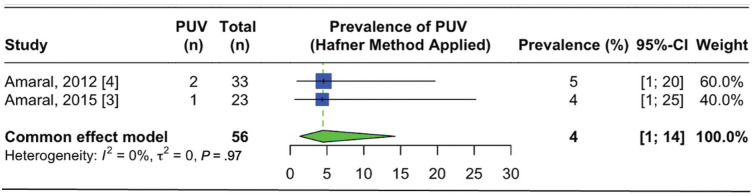
Meta-analysis of the prevalence of positive ulnar variance (PUV), assessed using the Hafner Method, in adolescent gymnasts.

Very low certainty of evidence from 2 studies^3,4^ that employed the Hafner Method for skeletally immature patients (n = 56 adolescent gymnasts) suggested a pooled prevalence of PUV of 4% (95% CI, 1%-14%; *I*^2^ = 0%) (Table 2, Appendix Figure A7). Across all gymnasts in these studies, the mean age was 11 ± 0.05 years, and the mean ulnar variance (UV) was −3 ± 2 mm. The remaining studies employed the method of perpendiculars^10^ or the concentric circle technique,^12^ both of which are more appropriate for skeletally mature individuals. Respectively, these studies reported PUV prevalence rates of 46% (mean age, 14 ± 2 years; mean UV, +0.5 ± 2 mm) and 71% (mean age, 16 years [SD not reported]; mean UV, +2 ± 1 mm). A single study among 25 gymnasts aged 10 ± 2 years reported that 16% acquired PUV after 18 months.^2^

### Wrist Pain Risk Factors

Four studies^16,18,20,22^ reported the risk factors associated with wrist pain in adolescent gymnasts (Table 3); however, their findings could not be meta-analyzed due to insufficient data. Multiple factors related to wrist pain were investigated including age, age at training onset, training intensity, BMI, years of training, and weekly training hours. Risk factors related to the chronological age of gymnasts were cited most frequently.^16,18,20^ Only 1 study^18^ reported the adjusted odds ratio (AOR) for significant risk factors among gymnasts including age >10 years (AOR, 16.28), age <14 years (AOR, 208.51), older age at training onset (AOR, 1.97), and increased intensity of training (AOR, 1.19) (defined as the product of training hours per week and competitive level grouping). Based on these findings, the risk for wrist pain was suggested to be significantly increased in gymnasts between 10 and 14 years old.^18^ Another study also reported increased BMI (OR, 1.72) and increased number of years training (OR, 1.02) as significant risk factors.^20^

**Table 3:** Significant Wrist Pain Risk Factors^*a*^

Author (y)	Type of Analysis Used	Risk Factor	Odds Ratio		*P*
			Crude	Adjusted	
DiFiori et al (2002)^16^	Multivariable logistic regression	Age (10-14 y)	NR	NR	.03
DiFiori et al (1996)^18^	Multivariable logistic regression	Age (>10 y)	NR	16.28	.02
		Age (<14 y)	NR	208.51	.02
		Increased age at training onset	NR	1.97	.02
		Increased training intensity	NR	1.19	.04
Fari et al (2021)^20^	Univariate logistic regression	Increased years of training (right wrist)	1.02	NR	.03
		Increased years of training (left wrist)	1.02	NR	.02
		Increased age (right wrist)	1.23	NR	.03
		Increased age (left wrist)	1.46	NR	.004
		Increased BMI (right wrist)	1.72	NR	.001
Guerra et al (2016)^22^	Chi-square	Increased weekly training hours	NR	NR	.04

a BMI, body mass index; NR, not reported.

In studies reporting on specific events,^3,15,16,18,22,41,50^ all gymnastics apparatuses (floor, pommel horse, balance beam, vault, parallel bars, horizontal bars, uneven bars, rings) were reported to cause wrist pain or injury (Table 1). However, the floor was the most reported apparatus used by both sexes to cause pain or injury among the studies.^15,16,18,22,41,50^ The pommel horse^3,15,16,18,22,50^ and balance beam^15,16,41,50^ were the most reported male-only and female-only apparatuses, respectively.

## Discussion

The major finding of this systematic review and meta-analysis of 25 studies was that young gymnasts demonstrated an alarmingly high prevalence of wrist pain and wrist injury. The highest risk identified was in 10- to 14-year-olds, which corresponds to the adolescent growth spurt. Wrist pain occurred in 53% of adolescent artistic gymnasts with a proportion of new cases of 2% over 1 year. Acute wrist injury occurred in 34% with a proportion of new cases of 4% over a median 2-year period, and chronic wrist injury occurred in 36% of adolescent gymnasts with a proportion of new cases of 5% over a median 2-year period. This study also found that PUV occurred in 4%, with a proportion of new cases of 16% over an 18-month period in preteen gymnasts.

While this study failed to establish a direct relationship between rates of distal radial physeal stress injury (GW) and PUV, it has previously been reported that GW can result in a 10% growth disturbance rate with 3% of those affected requiring surgical correction.^25^ Despite the acknowledgement for such consequences in the literature, this review identified a lack of consistent and specific reporting of the GW diagnosis across included studies. This makes it impossible to determine the true pooled rates of related complications such as pain or permanent skeletal changes.

The current review demonstrated that ages between 10 and 14 years, as well as increased age at training onset, training intensity, BMI, years of training, and weekly training hours were significant risk factors for wrist pain development in adolescent gymnasts. Risk factors related to the chronological age of gymnasts were cited most frequently.^16,18,20^ Only 1 study^18^ quantified the AOR for this variable and observed gymnasts between 10 and 14 years old to be at increased risk. This age cohort coincides with the onset of the adolescent growth period and encompasses the period of peak growth velocity (PGV), which occurs around 11 to 12 years in girls and 13 to 15 years in boys^51,53^ and reflects the maximal growth during the midgrowth spurt.^51^ During this period, alterations in the rate of mineralization cause temporary weakness, making the distal radial physis increasingly susceptible to injury.^18^ Repeatedly exposing the wrist to high-impact loads during the vulnerable PGV period may explain the relationship between age and gymnasts’ increased risk for wrist pain. Microtraumatic changes to the distal radial physis that result from these repetitive, high-impact wrist loads may also explain the association of wrist pain/injury with increased training intensity, increased BMI, more years of training, and longer weekly training hours.^26^ Furthermore, gymnasts who initiate their training at a later age may be closer to/within this vulnerable growth period, possibly increasing the risk for wrist pain/injury that is a direct result of GW.^18^

### Future Recommendations

Adolescent gymnasts experience high-intensity training during the period of peak skeletal growth.^13^ This highlights the need for evidenced-based SafeSport guidelines that optimize long-term wrist health by minimizing injury and permanent growth disturbances. Prospective studies using validated and systematically collected outcome measures are recommended to improve the epidemiological understanding of diagnosis-specific risk factors and to clarify the influence of training volume and training intensity on pain or injury. These are necessary to inform future injury surveillance tools and establish parameters for risk reduction guidelines. Finally, studies that examine other biomechanical and morphological factors suggested to influence wrist pain and injury risk, such as wrist braces, soft mats, and joint angles,^9,24,39,41^ will further aid in developing guidelines that are both relevant and informed.

### Strengths and Limitations

The main strengths of this study include the conducting of a comprehensive search and applying of explicit eligibility criteria, which allowed for inclusion of more studies in the current review compared with previous related reviews,^8,30^ as well as pooling of key outcomes. This review, however, was limited by the inclusion of studies with high risk of bias, as well as by heterogeneous definitions of pain/injury, study designs, and reported outcomes. Many studies reporting risk factors failed to present odds ratios or report on other athlete-specific and external factors previously suggested to influence GW risk.^53^ Last, the failure to discriminate among specific wrist injury diagnoses including GW, as well as a general tendency for gymnasts to underreport wrist pain or injury^42,47^ and overconformity to sport ethics, may all contribute to an underrepresentation of the true prevalence and incidence of the reported outcomes included in this study.

## Conclusion

Our review demonstrated that adolescent artistic gymnasts demonstrate an alarmingly high prevalence of acute and chronic wrist pain and wrist injury. Further research on training intensity thresholds and risk reduction strategies is imperative for informing and implementing guidelines that protect the health of this vulnerable population.

## Footnotes

†† References 3-5, 10, 12, 15, 17, 18, 22, 29, 43, 46, 48, 50.

‡‡ References 2-5, 7, 12, 15-18, 20, 22, 29, 31, 35, 37, 41, 43, 48, 50.

§§ References 2, 3, 5, 7, 10, 12, 15-18, 20, 22, 29, 31, 35, 37, 41, 46, 48, 50.

‖‖ References 2, 5, 7, 10, 12, 15-18, 20, 22, 31, 35, 41, 46, 48.

¶¶ References 3, 10, 15, 16, 18, 20, 29, 31, 35, 37, 50.

## References 3, 10, 15, 18, 20, 22, 35, 43, 50.

## Appendix

The following searches were conducted on February 2, 2024, and January 14, 2025.

**Table A1A:** Ovid MEDLINE Search Strategy

No.	Query	Results
1	((radial or radius) adj3 (epiphys* or growth plate* or physis or physeal)).tw,kf.	577
2	Wrist/	11,046
3	Radius/	10,591
4	epiphyses/ or growth plate/	11,874
5	(radius or radial or physis or physeal or wrist*).tw,kf.	196,121
6	or/1-5 [Wrist]	211,131
7	Gymnastics/	2389
8	(gymnast* or athlet* or sport*).tw,kf.	159,324
9	exp Sports/	220,632
10	or/7-9 [Athletes]	316,103
11	Fractures, Stress/	3934
12	musculoskeletal pain/	4746
13	Athletic Injuries/	31,284
14	((gymnast* or athlet* or sport*) adj5 (injur* or trauma* or strain* or fracture* or pain*)).tw,kf.	26,173
15	(gymnast* adj2 wrist*).tw,kf.	48
16	or/11-12 [Stress fractures general]	8670
17	or/13-14 [Athletic injuries]	46,589
18	16 and 10 [General stress fractures AND Athletes]	1915
19	17 or 18 [Athletes and athletic injuries combined]	47,548
20	6 and 19 [Wrist AND athletic injuries]	1827
21	15 or 20	1834
Additional results from January 14, 2025		99
Total Ovid MEDLINE results		1933

**Table A1B table5-23259671251395329:** Ovid EMBASE Search Strategy

#	Query	Results
1	((radial or radius) adj3 (epiphys* or growth plate* or physis or physeal)).tw,kf.	783
2	wrist/	40,301
3	radius/	17,485
4	epiphysis/ or epiphysis plate/	16,108
5	(radius or radial or physis or physeal or wrist*).tw,kf.	251,478
6	or/1-5 [Wrist]	274,603
7	gymnastics/	489
8	(gymnast* or athlet* or sport*).tw,kf.	204,640
9	exp sport/	227,960
10	or/7-9 [Athletes]	343,310
11	stress fracture/	8008
12	musculoskeletal pain/	15,813
13	sport injury/	36,688
14	((gymnast* or athlet* or sport*) adj5 (injur* or trauma* or strain* or fracture* or pain*)).tw,kf.	33,191
15	(gymnast* adj2 wrist*).tw,kf.	59
16	or/11-12 [Stress fractures general]	23,792
17	or/13-14 [Athletic injuries]	55,576
18	16 and 10 [General stress fractures AND Athletes]	3454
19	17 or 18 [Athletes and athletic injuries combined]	57,273
20	6 and 19 [Wrist AND athletic injuries]	2301
21	15 or 20	2309
Additional results from January 14, 2025		114
Total Ovid EMBASE results		2423

**Table A1C table6-23259671251395329:** CINAHL Search Strategy

#	Query	Results
S1	TI (((radial or radius) N3 (epiphys* or growth plate* or physis or physeal))) OR AB (((radial or radius) N3 (epiphys* or growth plate* or physis or physeal)))	107
S2	MH “Wrist”	3611
S3	MH “Radius”	1627
S4	MH “epiphyses”	1472
S5	TI (radius or radial or physis or physeal or wrist*) OR AB (radius or radial or physis or physeal or wrist*)	30,685
S6	S1 OR S2 OR S3 OR S4 OR S5	32,773
S7	MH “Gymnastics”	1079
S8	TI ((gymnast* or athlet* or sport*)) OR AB ((gymnast* or athlet* or sport*))	88,898
S9	MH “Sports+”	91,413
S10	S7 OR S8 OR S9	141,854
S11	MH “Fractures, Stress”	1937
S12	MH “musculoskeletal pain”	499
S13	MH “Athletic Injuries”	20,085
S14	TI (((gymnast* or athlet* or sport*) N5 (injur* or trauma* or strain* or fracture* or pain*))) OR AB (((gymnast* or athlet* or sport*) N5 (injur* or trauma* or strain* or fracture* or pain*)))	15,498
S15	TI (gymnast* N2 wrist*) OR AB (gymnast* N2 wrist*)	38
S16	S11 OR S12	2436
S17	S13 OR S14	28,723
S18	S10 AND S16	951
S19	S17 OR S18	29,033
S20	S6 AND S19	944
S21	S15 OR S20	949
Additional results from January 14, 2025		40
Total CINAHL results		989

**Table A1D table7-23259671251395329:** COCHRANE Search Strategy

#	Query	Results
1	((radial or radius) NEAR/3 (epiphys* or growth plate* or physis or physeal)):ti,ab,kw	9
2	[mh ^Wrist]	511
3	[mh ^Radius]	253
4	[mh ^epiphyses] or [mh ^”growth plate”]	26
5	(radius or radial or physis or physeal or wrist*):ti,ab,kw	16,910
6	{OR #1-#5}	16,930
7	[mh ^Gymnastics]	107
8	(gymnast* or athlet* or sport*):ti,ab,kw	22,926
9	[mh Sports]	21,906
10	{OR #7-#9}	38,141
11	[mh ^”Fractures, Stress”]	158
12	[mh ^”musculoskeletal pain”]	741
13	[mh ^”Athletic Injuries”]	968
14	((gymnast* or athlet* or sport*) NEAR/5 (injur* or trauma* or strain* or fracture* or pain*)):ti,ab,kw	3170
15	(gymnast* NEAR/2 wrist*):ti,ab,kw	0
16	{OR #11-#12}	899
17	{OR #13-#14}	3170
18	#16 AND #10	118
19	#17 OR #18	3260
20	#6 AND #19	81
21	#15 OR #20	81
Additional results from January 14, 2025		3
Total COCHRANE results		84

**Table A1E:** SPORTDiscus Search Strategy

#	Query	Results
S1	TI (((radial or radius) N3 (epiphys* or growth plate* or physis or physeal))) OR AB (((radial or radius) N3 (epiphys* or growth plate* or physis or physeal))) OR KW (((radial or radius) N3 (epiphys* or growth plate* or physis or physeal)))	64
S2	DE “WRIST”	1955
S3	DE "RADIAL bone"	268
S4	(DE "EPIPHYSIS") OR (DE "GROWTH plate")	610
S5	TI ((radius or radial or physis or physeal or wrist*)) OR AB ((radius or radial or physis or physeal or wrist*)) OR KW ((radius or radial or physis or physeal or wrist*))	12,421
S6	S1 OR S2 OR S3 OR S4 OR S5	12,990
S7	DE "GYMNASTICS"	11,993
S8	TI (gymnast* or athlet* or sport*) OR AB (gymnast* or athlet* or sport*) OR KW (gymnast* or athlet* or sport*)	665,845
S9	DE "SPORTS" OR DE "AERODYNAMICS in sports" OR DE "AERONAUTICAL sports" OR DE "AGE & sports" OR DE "AMATEUR sports" OR DE "ANIMAL sports" OR DE "ANTISEMITISM in sports" OR DE "AQUATIC sports" OR DE "BALL games" OR DE "BALLISTICS in sports" OR DE "BASEBALL" OR DE "BIOMECHANICS in sports" OR DE "COLLEGE sports" OR DE "COMBAT sports" OR DE "COMMUNICATION in sports" OR DE "CONTACT sports" OR DE "CROSS-training (Sports)" OR DE "DISC golf" OR DE "DISCRIMINATION in sports" OR DE "DOG sports" OR DE "DOPING in sports" OR DE "ENDURANCE sports" OR DE "EXTREME sports" OR DE "FANTASY sports" OR DE "FASCISM & sports" OR DE "FEMINISM & sports" OR DE "GAELIC games" OR DE "GAY Games" OR DE "GOODWILL Games" OR DE "GYMNASTICS" OR DE "HOCKEY" OR DE "HOMOPHOBIA in sports" OR DE "HYDRODYNAMICS in sports" OR DE "INDIVIDUAL sports" OR DE "KINEMATICS in sports" OR DE "KNIFE throwing" OR DE "LGBTQ+ people & sports" OR DE "LOG-chopping (Sports)" OR DE "MASCULINITY in sports" OR DE "MASS media & sports" OR DE "MILITARY sports" OR DE "MINORITIES in sports" OR DE "MOTION pictures in sports" OR DE "MOTORSPORTS" OR DE "NATIONAL socialism & sports" OR DE "NATIONALISM & sports" OR DE "NONVERBAL communication in sports" OR DE "OLYMPIC Games" OR DE "PARKOUR" OR DE "PHYSICS in sports" OR DE "PRESIDENTS – Sports" OR DE "PROFESSIONAL sports" OR DE "PROFESSIONALISM in sports" OR DE "RACISM in sports" OR DE "RACKET games" OR DE "RADAR in sports" OR DE "RECREATIONAL sports" OR DE "REGIONALISM & sports" OR DE "ROBOTICS in sports" OR DE "RODEOS" OR DE "ROLLER skating" OR DE "SCHOOL sports" OR DE "SENIOR Olympics" OR DE "SEXUAL harassment in sports" OR DE "SHOOTING (Sports)" OR DE "SHUTOUTS (Sports)" OR DE "SKATEBOARDING" OR DE "SOCIALISM & sports" OR DE "SOFTBALL" OR DE "SPORT for all" OR DE "SPORTS & state" OR DE "SPORTS & technology" OR DE "SPORTS & theater" OR DE "SPORTS & tourism" OR DE "SPORTS for children" OR DE "SPORTS for girls" OR DE "SPORTS for older people" OR DE "SPORTS for people with disabilities" OR DE "SPORTS for youth" OR DE "SPORTS forecasting" OR DE "SPORTS in antiquity" OR DE "SPORTS penalties" OR DE "SPORTS photography" OR DE "SPORTS rivalries" OR DE "SPORTS teams" OR DE "SPORTS tourism" OR DE "STEREOTYPES in sports" OR DE "TARGETS (Sports)" OR DE "TEAM sports" OR DE "TEAMWORK (Sports)" OR DE "TELEVISION & sports" OR DE "TRACEURS" OR DE "VIDEO tapes in sports" OR DE "VIOLENCE in sports" OR DE "WINTER sports" OR DE "WOMEN’S sports"	287,709
S10	S7 OR S8 OR S9	756,330
S11	DE "STRESS fractures (Orthopedics)"	1809
S12	TI (musculoskeletal N5 pain*) OR AB (musculoskeletal N5 pain*) OR KW (musculoskeletal N5 pain*)	1982
S13	DE "SPORTS injuries"	11,298
S14	TI (((gymnast* or athlet* or sport*) N5 (injur* or trauma* or strain* or fracture* or pain*))) OR AB (((gymnast* or athlet* or sport*) N5 (injur* or trauma* or strain* or fracture* or pain*))) OR KW (((gymnast* or athlet* or sport*) N5 (injur* or trauma* or strain* or fracture* or pain*)))	24,934
S15	TI gymnast* N2 wrist* OR AB gymnast* N2 wrist* OR KW gymnast* N2 wrist*	54
S16	S11 OR S12	14,445
S17	S13 OR S14	31,230
S18	S10 AND S16	3245
S19	S17 OR S18	33,027
S20	S6 AND S19	929
S21	S15 OR S20	914
Additional results from January 14, 2025		30
Total SPORTDiscus results		944

**Table A2:** Description of Outcomes Reported in Included Studies^*a*^

Author (y)	Outcome Name	Assessment Method	Follow-up, y	Definition
Albright et al (2023)^1^	Proportion of new cases—acute injury	Clinical exam	8	Reports of wrist sprain
Amaral et al (2015)^3^	Prevalence of PUV, WP	PUV: radiograph WP: questionnaire		PUV: Hafner Method^56^ criteria WP: presence of WP at time of data collection
Amaral et al (2014)^2^	Proportion of new cases—PUV	Radiograph	1.5	Hafner Method^56^ criteria
Amaral et al (2012)^4^	Prevalence of PUV	Radiograph		Hafner Method^56^ criteria
Aubergé et al (1984)^5^	Prevalence of chronic injury	Radiograph		Presence of sclerosis, flattened internal part of epiphysis, premature absence of physis, or condensation of subchondral bone or metaphysis
Caine et al (1992)^7^	Prevalence of chronic injury	Radiograph		Roy et al^41^ criteria
Chang et al (1995)^10^	Prevalence of PUV, WP	PUV: radiograph WP: questionnaire		PUV: Method of perpendiculars criteria^57^ WP: definition not provided
DeSmet et al (1994)^12^	Prevalence of PUV, chronic injury	PUV: radiograph Injury: radiograph		PUV: Concentric circle technique^58^) criteria Chronic injury: Carter et al^9^ criteria
DiFiori et al (2002)^16^	Proportion of new cases—WP; RFs	WP: questionnaire	1	WP: Any WP in the past 6 months of training
DiFiori et al (2002)^15^	Prevalence of WP	Questionnaire		Presence of WP in the past 6 months
DiFiori et al (1997)^17^	Prevalence of chronic injury	Radiograph		Roy et al^41^ and Carter et al^9^ criteria
DiFiori et al (1996)^18^	Prevalence of WP; RFs	WP: questionnaire		WP: Presence of WP in the past 6 months
Fari et al (2021)^20^	Prevalence of WP; RFs	WP: questionnaire		WP: Gymnastics-related pain involving muscles, tendons, joints, not resultant from specific traumas
Guerra et al (2016)^22^	Prevalence of WP, chronic injury; RFs	WP: questionnaire Injury: radiograph		WP: presence of WP at time of collection Chronic injury: Roy et al^41^ criteria
Konrad and Pförringer (1997)^29^	Prevalence of acute injury	Questionnaire		Gymnastics-related event resulting in loss of ≥1 week of training or medical treatment
Lindner and Caine (1990)^31^	Proportion of new cases—acute or chronic injury	Questionnaire	3	Sudden (acute) or gradual (chronic) onset injury causing inability to perform an activity for >1 day
McLaren et al (2015)^35^	Prevalence of WP	Questionnaire		Presence of current or recent history of WP
O’Kane et al (2011)^37^	Proportion of new cases—acute or chronic injury	Questionnaire	1	Acute: sudden onset injury preventing participation for ≥1 day Chronic: pain caused by gymnastics lasting ≥2 weeks, not resulting from acute injury
Roy et al (1985)^41^	Prevalence of chronic injury	Radiograph		Presence of widened distal radial physis, cystic changes, beaking distal epiphysis, blurring of radiolucent physis area
Sastre-Munar et al (2022)^43^	Prevalence of WP	Questionnaire		Gymnastics-related pain involving muscles, tendons, joints, not resultant from specific traumas
Shih et al (1995)^46^	Prevalence of acute injury	MRI		Presence of vertical or horizontal distal radial fractures
Swenson et al (2012)^48^	Prevalence of acute injury	Clinical exam		Reports of wrist fracture
Tisano et al (2022)^49^	Proportion of new cases—acute injury	Clinical exam	7	Reports of wrist fracture or wrist sprain/strain
Trevithick et al (2018)^50^	Prevalence of WP	Questionnaire		Presence of current WP
Wood et al (2010)^55^	Prevalence of acute injury	Radiograph		Presence of distal radial or ulnar fracture

a MRI, magnetic resonance imaging; PUV, positive ulnar variance; RF, risk factor; WP, wrist pain.

**Table A3:** Risk-of-Bias Assessment for All Included Studies^*a*^

Author (y)	Is Source Population Representative of the General Population?	Is Assessment of Outcome Valid/Reliable?	Are There Few Missing Data?	Risk-of-Bias Summary
Albright et al (2023)^1^	LR	LR	LR	LR
Amaral et al (2015)^3^	LR	HR	LR	HR
Amaral et al (2014)^2^	HR	LR	LR	HR
Amaral et al (2012)^4^	LR	LR	LR	LR
Aubergé et al (1984)^5^	HR	LR	LR	HR
Caine et al (1992)^7^	HR	LR	LR	HR
Chang et al (1995)^10^	HR	HR	LR	HR
DeSmet et al (1994)^12^	HR	LR	LR	HR
DiFiori et al (2002)^16^	HR	HR	LR	HR
DiFiori et al (2002)^15^	HR	HR	LR	HR
DiFiori et al (1997)^17^	HR	LR	LR	HR
DiFiori et al (1996)^18^	HR	HR	LR	HR
Fari et al (2021)^20^	HR	HR	HR	HR
Guerra et al (2016)^22^	HR	LR	LR	HR
Konrad and Pförringer (1997)^29^	LR	HR	HR	HR
Lindner and Caine (1990)^31^	HR	HR	HR	HR
McLaren et al (2015)^35^	HR	HR	LR	HR
O’Kane et al (2011)^37^	LR	HR	HR	HR
Roy et al (1985)^41^	HR	LR	LR	HR
Sastre-Munar et al (2022)^43^	LR	LR	LR	LR
Shih et al (1995)^46^	HR	LR	LR	HR
Swenson et al (2012)^48^	HR	LR	LR	HR
Tisano et al (2022)^49^	LR	LR	LR	LR
Trevithick et al (2018)^50^	LR	HR	HR	HR
Wood et al (2010)^55^	LR	LR	LR	LR

a HR, high risk; LR, low risk.

**Table A4:** Risk-of-Bias Assessment for Studies Reporting Risk Factors^*a*^

Author (y)	Did Study Adjust for Potential Covariates?	Did Study Use an Analysis Method That Limits Bias?	Risk-of-Bias Summary
DiFiori et al. (2002)^16^	LR	LR	LR
DiFiori et al. (1996)^18^	LR	LR	LR
Fari et al. (2021)^20^	HR	HR	HR
Guerra et al. (2016)^22^	HR	HR	HR

a HR, high risk; LR, low risk.
